# Wenxin Keli for atrial fibrillation: Protocol for a systematic review and meta-analysis

**DOI:** 10.1097/MD.0000000000010390

**Published:** 2018-04-27

**Authors:** Zhuogen He, Minan Zheng, Pingchang Xie, Yuanping Wang, Xia Yan, Dingwei Deng

**Affiliations:** a The Second Affiliated Hospital of Guangzhou University of Chinese Medicine, Guangdong Provincial Hospital of Chinese Medicine; b The Second Clinical School of Guangzhou University of Chinese Medicine, Guangzhou, China.

**Keywords:** atrial fibrillation, protocol, systematic review, Wenxin Keli

## Abstract

**Background:**

Atrial fibrillation (AF) is a most common cardiac arrhythmia in clinical practice. In China, Wenxin Keli (WXKL) therapy is a common treatment for AF, but its effects and safety remain uncertain. This protocol is to provide the methods used to assess the effectiveness and safety of WXKL for the treatment of patients with AF.

**Methods:**

We will search comprehensively the 4 English databases EMBASE, the Cochrane Central Register of Controlled Trials (Cochrane Library), PubMed, and Medline and 3 Chinese databases China National Knowledge Infrastructure (CNKI), Chinese Biomedical Literature Database (CBM), and Chinese Science and Technology Periodical database (VIP) on computer on March 2018 for the randomized controlled trials (RCTs) regarding WXKL for AF. The therapeutic effects according to the sinus rhythm and p-wave dispersion (Pwd) will be accepted as the primary outcomes. We will use RevMan V.5.3 software as well to compute the data synthesis carefully when a meta-analysis is allowed.

**Results:**

This study will provide a high-quality synthesis of current evidence of WXKL for AF.

**Conclusion:**

The conclusion of our systematic review will provide evidence to judge whether WXKL is an effective intervention for patient with AF.

**PROSPERO registration number:**

PROSPERO CRD 42018082045.

## Abstract 2

Supplemental Digital Content is available in the text

## 1 Introduction

Atrial fibrillation (AF), a most common cardiac arrhythmia in clinical practice, increasing cardiovascular mortality, severity of heart failure, and the risk of thromboembolic events has gotten more and more attention.^[1,2]^ It is reported that the prevalence of AF is about 0.5% to 1.0% in the general population, and the incidence of AF increases progressively with age, which attacks 54 per 100,000 individuals every year.^[3]^ Therefore, AF has obtained great importance in social public health care. On the contrary, it also becomes a heavy social burden for patients with AF are often hospitalized and it is estimated that from 2000 to 2010, the rate of AF hospitalizations in the United States increased by 23%.^[4]^

Recommeded by ACCF/American Heart Association/HRS practice guidline, current strategies for AF management include rate control, rhythm control, and antithrombotic therapy.^[3]^ All are aimed to ease symptoms, prevent tachycardia cardiomyopathy and thromboembolic events, and improve quality of life for AF patients.^[5]^ At present, the treatment of AF is mainly antiarrhythmic drugs (AADs) and catheter ablation. However, the therapeutic effects of traditional antiarrhythmic medicines are far from satisfactory because of the high rate of arrhythmia recurrence and the potential pro-arrhythmia effect.^[6]^ In recent years, a number of published documents have evidenced catheter ablation make significant program in AF treatment; however, it has a high recurrence rate, and patients often need to undergo surgery again.^[7]^ Therefore, researchers, clinicians, and patients have been finding novel approaches with effectiveness and safety for AF treatment.

Many clinical observations currently indicated that complementary and alternative medicine (CAM) has similar antiarrhythmic effects with AADs, along with few adverse events.^[8–10]^ Traditional Chinese Medicine (TCM), as a part of CAM, has been applied for the prevention and treatment of arrhythmic disease in China over a thousand years.^[11]^ Wenxin Keli (WXKL) is a Chinese herb extract examined to be significant efficacy and safety for treating AF.^[12]^ WXKL is composed of 5 Chinese herbs extracts, Nardostachys chinensis Batal extract (NcBe), Codonopsis, Notoginseng, amber, and Rhizoma polygonate; it is the first state-sanctioned TCM-based AAD marked in the 2009 National Reimbursement Drug List. The present animal experiment studies^[13,14]^ demonstrated that WXKL inhibits and prevents ventricular arrhythmias and atrial arrhythmias via complicated antiarrhythmic mechanisms. Approximately 5 million patients in Asia are administrating WXKL as an AAD for treatment of a variety of cardiac arrhythmias.^[15]^

Although only 1 systematic review^[16]^ on the WXKL treatment for AF was published 5 years ago, plenty of high-quality randomized controlled clinical researches (RCTs) have been published for the past few years.^[17,18]^ Therefore, it is necessary to update the search and assessment to provide the up-to-date evidence for AF management. Our systematic review and meta-analysis will answer 2 clinical questions about the WXKL treatment for the disease: whether WXKL is more effective and safer than AADs or placeboes; and whether WXKL combined with AADs is more effective and safer than the conventional therapy alone.

## 2 Methods

### 2.1 Types of studies

All the RCTs of WXKL for the treatment of AF will be included.

### 2.2 Types of patients

Participants clinically diagnosed with AF,^[19]^ over 18 years old, will be included, without the limitation of gender, race, or the classification of AF.

### 2.3 Types of interventions

The therapy used in the experimental group is WXKL only for orally taking alone or combined with conventional AADs. The control group could be conventional medicine or placebo.

### 2.4 Types of outcome measures

#### 2.4.1 Primary outcomes

The primary outcome measure was maintenance of sinus rhythm and p-wave dispersion (Pwd).

#### 2.4.2 Secondary outcomes

**The secondary outcomes are as follows:**
• Bleeding events;
• Embolic events;
• Symptom improvement (such as chest distress etc);
• Frequency of PAF attack;
• Quality of life, such as QOL Scale.

### 2.5 Search methods for the identification of studies

#### 2.5.1 Electronic searches

We will search comprehensively the 4 English databases EMBASE, the Cochrane Central Register of Controlled Trials (Cochrane Library), PubMed, and Medline and 3 Chinese databases China National Knowledge Infrastructure (CNKI), Chinese Biomedical Literature Database (CBM), and Chinese Science and Technology Periodical database (VIP) on computer in March 2018 for the RCTs regarding WXKL for AF. According to the instruction of Cochrane handbook, we made detailed strategies for searching the PubMed database in Appendix A and similar strategies will be applied to remaining databases.

#### 2.5.2 Searching other resources

At the same time, a list of medical journals in Guangzhou University of Chinese Medicine libraries will be searched as a supplement, such as *Journal of Traditional Chinese Medicine*.

### 2.6 Data collection and analysis

#### 2.6.1 Selection of studies

**Figure 1:**
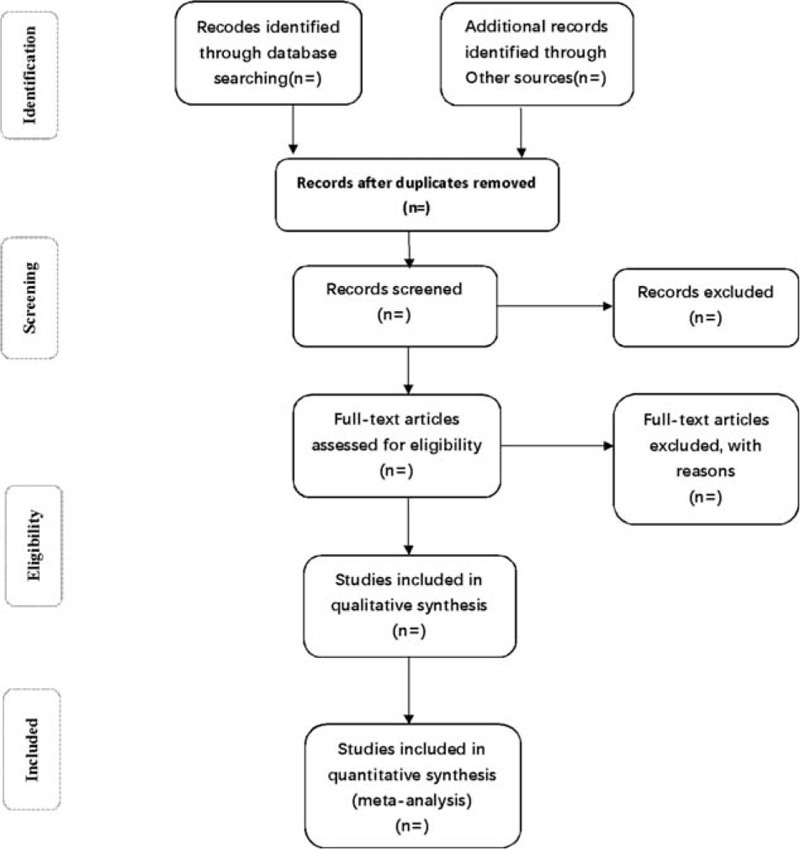
Flow diagram of study selection process.

Two professional researchers will import papers into Endnote X7 the research and reference manager and retrieve potential articles through titles, abstracts, and full texts in order to screen eligible literatures, according to the inclusion criteria and exclusion criteria. Both searching and the screening will be performed by the 2 reviewers independently. Any uncertain or missed data will be clarified by contacting the author for the details. We will also solve disagreement in discussion with the third author. The specific process of articles selection is shown in a Preferred Reporting Items for Systematic Review and Meta-analysis (PRISMA) flow diagram (Fig. 1).

#### 2.6.2 Data collection and management

Another 2 researchers will extract the basic data and outcome data of included literatures independently according to the data management table designed in advance: the included trials, first author, journal source, publication time, research design and key elements of quality evaluation, detail information regarding the treatment and control group, outcome indicators, relevant indicators of bias risk assessment, and adverse events. Eventually, another experienced member will deal with the inconsistencies.

#### 2.6.3 Assessment of risk of bias in included studies

Two independent reviewers will be respondent to appraise the risk of bias about eligible studies, on the basis of Cochrane Handbook for Systematic Reviews of Interventions V.5.1.0. If any disagreement is there in assessment, we will reach a consensus via group discussion or consulting with a senior author if necessary. The following 7 domains will be evaluated for the risk of bias, random sequence generation, allocation concealment, blinding of participants and personnel, blinding of outcome assessment, incomplete, outcome data, selective reporting, and other bias. Ultimately, the assessment will be classified into 3 grades: “low risk of bias,” “high risk of biases,” or “unclear risk of bias.”

#### 2.6.4 Measures of treatment effect

The review will use RevMan 5.3 (Version 5.3, Copenhagen: The Nordic Cochrane Center, The Cochrane Collaboration, 2014) to compute meta-analysis when the collected data are available. For dichotomous outcomes, the rate ratio (RR) will be conducted to indicate extracted data. For measurement data, the mean difference (MD) will be employed correspondingly to data synthesis. A 95% confidence interval (CI) will be adopted in either RR or MD to express the effect sizes.

#### 2.6.5 Dealing with missing data

If the required data are not clear or not reported in clinical papers, the reviewers will connect with the original author of the studies via e-mail for complete information. If not, we will analyze available data to perform the outcome; in the meanwhile, we will also assess the potential impact the missing data might cause on the conclusion in the discussion.

#### 2.6.6 Assessment of heterogeneity

Heterogeneity between trials of included studies will be evaluated by *I*^*2*^. In the case that *I*^*2*^ in primary outcome is over 50%, we make conclusion that statistical heterogeneity is significant and conduct descriptive statistical analysis for data synthesis and the subgroup analysis will be performed to detect the potential factors. Conversely, when *I*^*2*^ less than 50%, it is considered low heterogeneity and to use a Chi-squared test to investigate statistical heterogeneity.

#### 2.6.7 Assessment of reporting bias

If included trials are exceed 10 in the review, we will make visual asymmetry on a funnel plot via Egger methods in order to detect reporting biases or small-study effects.

#### 2.6.8 Data synthesis

RevMan software will be used to conduct the data analysis when the evidence is reliable and complete that a meta-analysis is suitable. When *I*^*2*^<50%, RR and MD will be computed via the fixed effects model; otherwise, data synthesis will be presented in using the random-effects model. If apparent clinical heterogeneity is demonstrated, the reviewers can carry out the subgroup or sensitivity analysis to explore heterogeneity source including clinical and methodology cause. On the contrary, we only perform descriptive analysis if meta-analysis is not applicable.

#### 2.6.9 Subgroup analysis

Subgroup analysis will be generated if the eligible studies are sufficient (at least 10 trials). With the purpose to explore the resources of the heterogeneity, we will take inconsistent participants characteristic, classification of AF, disease course, types of intervention (WXKL or WXKL combined with conventional AADs), frequency of taking medicine, and other unpredictable factors into account.

#### 2.6.10 Sensitivity analysis

If it is possible, we will proceed a sensitivity analysis to test the robustness of the conclusion, for example, reconduct a meta-analysis and compare with the original one after removing the low quality or small size trial, to explore whether these factors influence the total effect of meta-analysis.

#### 2.6.11 Ethics and dissemination

This meta-analysis does not need ethical approval because there are no data used in our study that are linked to individual patient data. Also, the findings will be disseminated through a peer-review publication.

## 3 Discussion

TCM WXKL is a potentially effective, having less side effects CAM for AF patients, though the mechanism remains poorly clear and needs to be explored further.^[16]^ To our knowledge, whether WXKL is effective and safe on AF has not been clearly demonstrated. Therefore, we conduct the review aiming to provide a more leading-edge and objective evidence for clinicians. More and more AF patients may also benefit from potential alternative interventions. The systematic review is composed of identification, research contents, data extraction, and date synthesis. However, the meta-analysis conclusion may be affected by some potential limitations. First, the strategies of electronic searches are limited by Chinese or English, those potential articles published in non-English and non-Chinese, such as Japanese, Korean, or German cannot be included, causing selection bias. In addition to conceptual design, it is hard to conduct blinding in both participants and researchers in original trials may lead to the high risk of bias.

## Author contributions

**Conceptualization:** Dingwei Deng, Zhuogen He, Yuanping Wang.

**Data curation:** Dingwei Deng, Zhuogen He, Yuanping Wang.

**Formal analysis:** Pingchang Xie.

**Investigation:** Xia Yan.

**Methodology:** Minan Zheng.

## Supplementary Material

Supplemental Digital Content
