# Supplementary material for: Wenxin Keli for atrial fibrillation: Protocol for a systematic review and meta-analysis
Source: Medicine (Baltimore). 2018 Apr 27;97(17):e0390. doi: 10.1097/MD.0000000000010390 (PMC5944514; doi:10.1097/MD.0000000000010390)
Supplement: Supplemental Digital Content [file medi-97-e0390-s001.docx]

**Appendix A.**

***Search strategy used in PubMed database***

#1 wenxin keli

#2 Atrial Fibrillations OR Fibrillation, Atrial OR Fibrillations, Atrial OR Auricular Fibrillation OR Auricular Fibrillations OR Fibrillation, Auricular OR Fibrillations, Auricular OR Persistent Atrial Fibrillation OR Atrial Fibrillation, Persistent OR Atrial Fibrillations, Persistent OR Fibrillation, Persistent Atrial OR Fibrillations, Persistent Atrial OR Persistent Atrial Fibrillations OR Familial Atrial Fibrillation OR Atrial Fibrillation, Familial OR Atrial Fibrillations, Familial OR Familial Atrial Fibrillations OR Fibrillation, Familial Atrial OR Fibrillations, Familial Atrial OR Paroxysmal Atrial Fibrillation OR Atrial Fibrillation, Paroxysmal OR Atrial Fibrillations, Paroxysmal OR Fibrillation, Paroxysmal Atrial OR Fibrillations, Paroxysmal Atrial OR Paroxysmal Atrial Fibrillations

#3 Randomized controlled trial OR clinical study OR Clin-ical Trial OR Controlled study OR Controlled Trial OR Random*Control* study OR random* Control* Trial

#1 AND #2 AND #3
